# Nutritional Status and Bone Microarchitecture in a Cohort of Systemic Sclerosis Patients

**DOI:** 10.3390/nu12061632

**Published:** 2020-06-01

**Authors:** Sabrina Paolino, Greta Pacini, Carlotta Schenone, Massimo Patanè, Alberto Sulli, Samir Giuseppe Sukkar, Adriano Lercara, Carmen Pizzorni, Emanuele Gotelli, Francesco Cattelan, Federica Goegan, Vanessa Smith, Maurizio Cutolo

**Affiliations:** 1 Research Laboratory and Academic Division of Clinical Rheumatology, Department of Internal Medicine DiMI, University of Genoa, IRCCS San Martino Polyclinic, 16126 Genoa, Italy; sabrina.paolino@unige.it (S.P.); greta.pacini@gmail.com (G.P.); carlottaschenone@gmail.com (C.S.); dr.massimo.patane@gmail.com (M.P.); albertosulli@unige.it (A.S.); adriano.lercara@gmail.com (A.L.); carmen.pizzorni@unige.it (C.P.); emanuele.gotelli@live.it (E.G.); francesco.cattelan.1991@gmail.com (F.C.); federica_188@yahoo.it (F.G.); 2 Clinical Nutrition Unit, IRCCS San Martino Polyclinic, 16132 Genoa, Italy; samir.sukkar@hsanmartino.it; 3 Department of Internal Medicine, Ghent University, St. Pietersnieuwstraat 33, 9000 Gent, Belgium; 4 Department of Rheumatology, Ghent University Hospital, Corneel Heymanslaan 10, 9000 Gent, Belgium; 5 Unit for Molecular Immunology and Inflammation, VIB Inflammation Research Center (IRC), 9000 Ghent, Belgium

**Keywords:** systemic sclerosis, malnutrition, bone parameters

## Abstract

Systemic sclerosis (SSc) is a connective tissue disease characterized by initial microvascular damage, immune system activation and progressive fibrosis with insufficiency of internal organs. Gastrointestinal (GI) involvement is characterized by atrophy of the smooth muscle and small bowel hypomotility, mainly resulting from an autonomic nerve dysfunction. These modifications significantly affect gut transit and nutrient absorption, thus leading to malnutrition deficit induced by malabsorption. Nutritional deficit induced by malabsorption might also lead to bone alterations. This study aims to evaluate the relationship between malnutrition and bone status. Thirty-six postmenopausal female patients fulfilling the ACR 2013 criteria for SSc underwent dual-energy X-ray absorptiometry scan (DXA) to detect quantitative lumbar spine bone mineral density (BMD) and trabecular bone score (TBS) analysis to detect bone quality. Data from DXA also allow to assess body composition and provide several quantitative parameters, including free fat mass index (FFMI) that identifies the patient with malnutrition (values <15 kg/m^2^ in women and 17 kg/m^2^ in men), according to the ESPEN criteria. Body mass index (BMI) was calculated for all SSc patients and every patient completed a diary reporting GI symptoms. Two groups of SSc patients with or without diagnosed malnutrition according to FFMI parameter were identified. Malnourished SSc patients showed significantly lower weight (*p* = 0.01) and BMI (*p* = 0.001), as well as lower serum levels of hemoglobin (*p* = 0.009), albumin (*p* = 0.002), PTH (*p* = 0.02) and 25OH-vitamin D (*p* = 0.008). DXA analysis showed significantly lower lumbar L1-L4 T-score (*p* = 0.009) and BMD values (*p* = 0.029) in malnourished SSc patients. Consistently, TBS values were significantly lower in malnourished patients (*p* = 0.008) and correlated with BMD (at any site) and serum albumin levels (*p* = 0.02). In addition, FFMI positively correlated with bone parameters as well as with symptoms of intestinal impairment in malnourished SSc patients. Finally, GI symptoms significantly correlated with BMD but not with TBS. This pilot study shows that in malnourished SSc patients (2015 ESPEN criteria: FFMI<15 kg/m^2^), an altered bone status significantly correlates with GI involvement, in terms of symptoms being mainly due to intestinal involvement together with the presence of selected serum biomarkers of malnutrition.

## 1. Introduction

Systemic sclerosis (SSc) is an autoimmune connective tissue disease characterized by early microvascular damage, immune activation and progressive irreversible fibrosis of skin and internal organs [1,2].

Gastrointestinal tract (GI) involvement is frequent in SSc patients, regardless of belonging to the diffuse cutaneous SSc subset (dcSSc) or the limited cutaneous SSc subset (lcSSc) [3,4,5].

Although any part of the GI tract can be affected, 90% of patients report symptoms related to upper GI tract involvement and 40% of patients report symptoms related to lower GI involvement [6,7]. GI tract dysfunctions are sustained by diffuse vascular abnormalities. This leads to ischemia and immunological events and to the conversion of fibroblasts into myofibroblasts, which produce an excessive amount of extracellular matrix proteins, thus inducing fibrosis and atrophy of smooth muscle and distortion of the normal tissue architecture. These mechanisms, in addition to autonomic nerve dysfunction sustained by anti M3-R antibodies activation, may contribute to GI dysmotility [8,9,10].

In SSc, small intestinal motility dysfunction is reported in up to 88% of patients [6]. Intestinal dysmotility promotes stasis of lumen content resulting in over-bacterial colonization, which also seems to be determined using proton pump inhibitors (PPI) and consequent achlorhydria [6]. These mechanisms may contribute to intestinal malabsorption and thus high risk of malnutrition [6,7,8,9,11,12].

Through the gut epithelium, nutritional deficiencies caused by malabsorption interfere with the transfer of macro and micronutrients necessary for bone metabolism. Thus, malabsorption, in addition to systemic inflammation, may increase the risk of secondary osteoporosis (OP) [13]. Previous studies reported low values of bone mass density (BMD), thus finding a higher incidence of OP and increased risk of fractures in SSc patients [14,15]. Very little is known about bone quality and no specific relationship has emerged between GI involvement and bone health in this setting of SSc patients [16].

The aim of this study was to evaluate the relationship between markers of malnutrition and bone microarchitecture quality, detected by TBS, according to symptoms of GI involvement in a cohort of SSc patients.

## 2. Materials and Methods

In this retrospective study, 36 consecutive postmenopausal SSc female patients, followed at Scleroderma Clinic of the Rheumatology Division of Genova University (Italy), were recruited. All patients and controls were aged over 18 years. The diagnosis of SSc was based on the 2013 American College of Rheumatology (ACR)/European League against Rheumatology (EULAR) classification criteria [17].

Patients with medical history of malignancies or overlap with other autoimmune diseases (such as Rheumatoid Arthritis, Sjögren’s Syndrome, Systemic Lupus Erythematosus and inflammatory myopathies) or with other possible causes of OP (such as severe neurological, pulmonary, cardiac and endocrine diseases or osteopenic drugs such as glucocorticoids) were not included. The study was conducted in accordance with the principles of Good Clinical Practices and the declaration of Helsinki. All patients provided the mandatory signed informed consent form at the right time to enter the Scleroderma Clinic assistance. All the standard clinical investigations performed at the Scleroderma Clinic were approved by the local Ethical Board Committee (EBC).

All SSc patients underwent clinical evaluation, laboratory and instrumental exams as part of a regular follow-up approved by SSc international guidelines and local clinical practice (EBC) [18].

Demographic and lifestyle data included: sex, age, disease duration (defined since the first non-Raynaud phenomenon symptom that satisfies the 2013 ACR/EULAR criteria), smoking condition, alcohol consumption, prior fragility fractures, familiarity with femoral fractures, menopausal status, body weight and height (with body mass index—BMI), weight loss. Every patient’s ongoing treatment that could influence bowel motility, such as opioids, was recorded.

### 2.1. Laboratory Tests

Routine follow-up laboratory tests were performed (blood cell count, creatinine (crea), erythrocyte sedimentation rate (ESR), C reactive protein (CRP)). In addition, bone metabolism parameters, such as serum bone alkaline phosphatase (BAP), calcium (Ca), phosphorus (Ph), parathyroid hormone (PTH) and 25-hydroxyvitamin D (25OH)D, were included.

Among the blood tests performed, parameters of malnutritional status were included, so we considered hemoglobin (Hb) and serum albumin [19]. Antinuclear antibodies (ANAs) were assessed using indirect immunofluorescence on Hep-2/liver cells (EUROPLUS ANA Mosaic FA 1510-1), with a 1:80 serum dilution as a cut-off value. Extractable Nuclear Antigen antibodies (ENA) were assessed using ELISA (EUROASSAY Anti-ENA ProfilePlus ELISA IgG, EA 1590-1G).

### 2.2. Nutritional Assessment in SSc Patients

Both body composition parameters and bone density were determined by Dual X-ray absorptiometry (DXA scan, Lunar Prodigy, GE Lunar, Madison, WI, USA), performed by a single experienced technician.

A dedicated software, by non-invasive techniques, analyzed the whole body composition and the different body composition of three major areas (arms, legs and trunk), describing total body mass (TM) (gr), total lean mass (LM) (gr), total fat mass (FM) (gr) and bone mineral content (BMC—g/cm^2^) of each area.

The fat free mass index (FFMI) was derived from FFM (fat free mass) (kg) divided by height squared (m^2^) [20]. Malnutrition was assessed according to 2015 ESPEN criteria and defined as FFMI<15 kg/m^2^ in women and <17 kg/m^2^ in men [21].

### 2.3. Bone Assessment

Bone mineral density (BMD) was recorded classically at the lumbar spine (L1-L4) and femoral neck, also in other seven different body areas (head, upper limbs, lower limbs, trunk, spine, ribs, pelvis). According to the criteria recommended by WHO in 1994, OP was diagnosed by a T-score < −2.5 × standard deviation (SD) at any site on the lumbar spine, femoral neck, or total hip [22].

The trabecular bone score (TBS) was indirectly acquired at the same region of the BMD measurement and the value was expressed as the average of L1-L4 evaluation. This tool measures the pixel variations that correspond to the attenuation of the X-ray that crosses each single point of the vertebra. The result reflects the absorption of beams of ray in grayscale and represents different microarchitectural 3D conformations of the bone trabecular tissue (TB). A cut-off was proposed in postmenopausal women to identify normal and abnormal microarchitecture: TBS = 1.350 or more (normal); 1.200 < TBS > 1.350 (partially degraded); and a TBS of 1.200 or less (degraded) [23,24].

Data regarding fragility fractures of hip and non-vertebral non-hip sites were recorded by an anamnestic questionnaire. At the same time as DXA, all SSc patients underwent the vertebral fracture assessment (VFA) using a software incorporated in DXA scan, Lunar Prodigy, GE Lunar, Madison, WI, USA, to detect fragility in vertebral fracture at the dorsal-lumbar spinal tract [25].

### 2.4. Gastrointestinal Involvement

To explore GI symptoms, a questionnaire recording the manifestations of intestine involvement was administered to each patient. The questionnaire reported the main symptoms of intestinal impairment, such as postprandial bloating, abdominal distension, abdominal pain, diarrhea [26].

### 2.5. Statistical Analysis

Statistical analysis was performed using Prism version 5.02 (GraphPad Software, La Jolla, CA, USA). Data were expressed as a frequency distribution or as a median and interquartile range (IQR). Chi squared and Fisher tests were performed to explore associations between categorical variables. Non-parametric Mann Whitney U- and Kruskall-Wallis tests were performed to compare continuous variable distributions between independent groups. Pearson’s and Spearman’s correlations were calculated to measure linear relationships between variables with continuous and ordinal distributions respectively; linear and logistic regressions were used to model relationships between variables. A *p* value <0.05 and a confidence interval (CI) of 95% were considered statistically significant.

## 3. Results

Clinical, laboratory and bone parameters according to the malnutrition assessment were recorded. There were no significant differences in the principal demographic data (age, height, disease duration) between the two groups of SSc patients (malnourished and not), based on FFMI malnutrition assessment (See Table 1).

In addition, no differences regarding the most important risk factors for OP, such as smoking condition, alcohol consumption, familiarity with hip fractures and previous OP-related fractures, were observed between the SSc patient groups.

Malnourished patients showed lower weight (*p* = 0.01) and BMI (*p* = 0.001). Regarding blood bone turnover markers, no significant abnormalities were observed in the median values of serum calcium (*p* = 0.59), phosphorus (*p* = 0.41) and bone alkaline phosphatase (*p* = 0.59) but significant differences were reported in the median values of PTH (*p* = 0.02) and 25OH vitamin D (*p* = 0.008).

Additionally, blood tests revealed significantly lower serum concentration of both hemoglobin and albumin levels in malnourished SSc patients (*p* = 0.009 and *p* = 0.002 respectively).

The analysis of bone status with dedicated tools revealed a lower lumbar L1-L4 T-score (*p* = 0.009) in malnourished patients and a further detailed evaluation of bone mass in different body areas showed a significantly lower BMD at the level of lumbar spine in SSc malnourished patients only (*p* = 0.03) (Table 1).

Of note, TBS was significantly lower in malnourished SSc patients (*p* = 0.008) and a positive correlation was observed between TBS and BMD values in arms (*p* = 0.036), legs (*p* = 0.01), total trunk (*p* = 0.05), pelvis (*p* = 0.04), total femur (*p* = 0.03). Additionally, TBS positively correlated with lumbar L1-L4 T-score (*p* = 0.04), total femur T-score (*p* = 0.01) and with malnutrition/malabsorption markers, such as serum albumin levels (*p* = 0.02). FFMI significantly correlated with bone density and bone quality parameters in a positive manner (Table 2).

Interestingly, in 53.8% of malnourished SSc patients, self-reported symptoms of intestinal impairment were recorded (bloating, nausea, vomiting, abdominal pain, diarrhea). In these patients, DXA showed significantly lower BMD at any site (BMD arms (*p* = 0.02), BMD legs (*p* = 0.01), BMD total trunk (*p* = 0.001), BMD ribs (*p* = 0.009), BMD lumbar spine (*p* = 0.007), BMD pelvis (*p* = 0.0002), BMD total femur (*p* = 0.002)) but no significant differences with TBS (*p* = 0.43).

## 4. Discussion

This study found a significant correlation between bone quality in terms of microarchitecture and nutritional status in malnourished SSc patients.

Malnutrition had already been reported as a possible complication of SSc with a negative impact on disease prognosis. Several methods for screening and diagnosis of malnutrition have been validated, still having a rather confused terminology, though. As a consequence, a comparison among different studies regarding the prevalence of malnutrition in SSc patients is difficult to perform. In fact, the reported malnutrition prevalence in SSc varies from 5.3% to 55.6%, depending on the different diagnostic criteria used [27,28].

In this study, we used the 2015 ESPEN criteria (FFMI<15 kg/m^2^) to identify malnourished patients and we reported a prevalence of 38.8%, in line with a previous large study [29].

Possible specific disease conditions that may increase the risk of malnutrition in SSc patients include microstomia, difficult mouth opening, sicca syndrome, reduced gastric motility and digital ulcers or sclerodactyly that limit feeding due to the altered hand ability in the advanced stages of the disease [11,30,31].

Notably, the GI tract is frequently affected in SSc patients, regardless of the extent of skin involvement. In particular, a prevalence rate of 40–88% of intestinal motility dysfunction was reported in SSc patients, 30% of them referring lower GI symptoms. Interestingly, a recent study showed that 23% of patients presented GI involvement without differences between dcSSc and lcSSc status [6,7,8,9,10,11,32].

In SSc disease, GI tract hypomobility results in a local stasis of ingested nutrients, which leads to altered microbiota (dysbiosis) and malabsorption, the latter promoting malnutrition and weight loss [12,32,33]. In our cohort, the prevalence of malnourished SSc patients presenting symptoms of intestinal impairment was 53.8%, in agreement with previous observations [27,28].

Local dysbiosis plays an integral role in the disrupted transportation and uptake of micronutrients (i.e., calcium and vitamin D) and macronutrients (such as proteins) needed for bone remodeling, thus predisposing individuals to osteomalacia or OP [34].

Nutrients are directly linked to bone density in total body, spine and hip. In particular, bone turnover changes are related to dietary intake of proteins: low proteins and low-serum albumin are strongly associated with higher hip fracture risk [35,36]. Our data confirm that albumin positively and significantly correlates with both bone mass (assessed by BMD at all the skeleton sites) and bone microarchitecture (assessed by TBS).

TBS represents a relative index of bone texture and provides information about bone microarchitectural quality, thus improving the assessment of fracture risk [37,38]. Interestingly, this study reports that TBS positively and significantly correlates with BMD of the whole femur and of other four sites of the skeleton in malnourished SSc patients. In a previous study, SSc patients showed lower values of TBS compared to healthy controls and similar correlations between TBS and BMD were observed [39].

Protein intake/content could positively influence bone status in terms of micro-skeleton protein composition, as confirmed in the present study, whereas TBS positively and significantly correlates with albumin serum concentration in SSc patients [40,41,42].

Previous literature already reported a significant reduction of serum albumin, Hb and 25(OH)D concentrations that are recognized malnutrition biomarkers [19,29,43].

In particular, recent data also support a critical role of vitamin D in maintaining integrity not only of skeletal health but also of the junction complexes of the bowel epithelium that allows mucosal barrier homeostasis [44]. Gut-local alterations in SSc patients with severe organ involvement contribute to the complex immune disturbances that are related to the prevalence of vitamin D deficiency/insufficiency compared to the general population [45,46]. In fact, gut is one of the extra-renal sites where inflammation induces local hyper-expression of CYP24A1 and CYP27B1—enzymes that decrease serum 25(OH)D concentration [46,47].

Patients with SSc are subject to an increased risk of OP and one of the largest cohorts in the literature showed 23.6% prevalence. In the present study, prevalence of OP at the lumbar spine (25%) was found in line with previous data (23.6%), with a higher prevalence in malnourished SSc patients (38.4%) [14,15].

Of note, present data from bone fractures revealed that 30.5% of SSc patients had fractures (19.4% vertebral fractures) and among these fractured patients, 13.8% were malnourished according to ESPEN criteria. Additionally, 41.6% of them also reported symptoms of intestinal impairment.

In line with recent studies, a significant lower bone mass was observed in malnourished patients, together with a decreased BMD in lumbar spine and lower T-score values, both at the femur and at the lumbar spine levels [14,15].

Our present investigation had some limitations, including: (a) a small number of participants, which limited the statistical investigations; (b) no specific validated questionnaire to investigate symptoms of small bowel involvement; (c) no comparison with the gold standard for SIBO diagnosis, so we could not provide the number of false positives and false negatives; (d) no evaluation of the possible role of cardiopulmonary involvement in cardiac cachexia; and (e) no multivariate analysis assessing the possible effect of confounding factors [48].

In conclusion, this study reports an intriguing association between bone and nutritional status, particularly in SSc patients with symptoms mainly related to bowel involvement. Further investigations are needed in order to better elucidate any clinically relevant association between body composition and SSc gastrointestinal complications.

## Figures and Tables

**Table 1:** Comparisons of bone parameters between malnourished and non-malnourished systemic sclerosis (SSc) patients.

	Malnutrition (Yes)	Malnutrition (No)	*p* Value
Patients, *n* (%)	13/36 (36.1%)	23/36 (63.8%)	
Age, median (IQR), years	54 (43–75)	66 (43–85)	0.13
Disease duration, median (IQR), months	222 (48–314)	230 (53–366)	0.2
Weight, median (IQR), kg	56 (48–67)	64.5 (47–84)	0.01
Height, median (IQR), cm	165 (154–175)	160 (151–180)	0.12
BMI, median (IQR), kg/m^2^	19.4 (17–26.2)	24.3 (20.3–30.5)	0.001
Smoke*, *n* (%)	3/13 (23%)	1/23 (4.3%)	0.08
Alcohol consumption**, *n* (%)	2/13 (15.4%)	5/23 (38.5%)	0.64
Previous osteoporosis related fractures, *n* (%)	5/13 (38.4%)	5/23 (38.5%)	0.28
Previous vertebral osteoporosis fractures, *n* (%)	3/13 (23%)	4/23 (17.4%)	0.67
Previous hip osteoporosis fractures, *n* (%)	0/13 (0%)	0/23 (0%)	0.64
Previous non-vertebral non-hip fractures, *n* (%)	3/13 (23%)	1/23 (4.3%)	0.08
Family history of hip fractures, *n* (%)	4/13 (30.7%)	2/23 (8.7%)	0.87
Vertebral osteoporosis, *n* (%)	5/13 (38.4%)	4/23 (17.4%)	0.16
Femoral osteoporosis, *n* (%)	1/13 (7.7%)	2/23 (8.7%)	0.91
lcSSC, *n* (%)	8/13 (61.5%)	18/23 (78.2%)	0.28
dcSSC, *n* (%)	5/13 (38.5%)	5/13 (38.5%)	0.63
mRSS (IQR)	13 (0–32)	10 (0–28)	0.61
**Bone Parameters**			
Arms BMD, median (IQR), g/cm^2^	0.715 (0.44–0.84)	0.715 (0.548–1.158)	0.88
Legs BMD, median (IQR), g/cm^2^	0.937 (0.784–1.11)	1.010 (0.684–1.506)	0.27
Lumbar spine L1-L4 BMD, median (IQR), g/cm^2^	0.916 (0.703–1.123)	1.013 (0.713–1.511)	0.03
Ribs BMD, median (IQR), g/cm^2^	0.630 (0.471–0.741)	0.688 (0.44–1.099)	0.16
Total trunk BMD, median (IQR), g/cm^2^	0.755 (0.645–0.882)	0.835 (0.560–1.220)	0.09
Pelvis BMD, median (IQR), g/cm^2^	0.770 (0.652–0.993)	0.861 (0.524–1.231)	0.05
Total femur BMD, median (IQR), g/cm^2^	0.941 (0.825–1.144)	1.051 (0.724–1.39)	0.29
L1-L4 T-score, median (IQR)	−2.3 (−4.3; −0.3)	−0.8 (−3.1; −2)	0.009
Total femur T-score, median (IQR)	−1.2 (−2.5; 0.6)	−0.5 (−3.5; 2.4)	0.14
TBS, median (IQR)	1087 (1043–1366)	1183 (0.08–1348)	0.008
**Laboratory Tests**			
Hb, median (IQR), g/dL	11.6 (10.6–13.1)	12.5 (10.6–13.1)	0.009
25(OH)D, median (IQR), ng/mL	18.3 (4.6–41.3)	29.7 (9.3–37.2)	0.008
Ca, median (IQR), mg/dL	9.6 (9–10)	9.5 (8.1–10.2)	0.59
Ph, median (IQR), mg/dL	3.5 (2.9–4.3)	3.3 (2.3–4)	0.41
PTH, median (IQR), ng/L	18 (12–34)	27 (12–75)	0.02
ALP-b, median (IQR), µg/L	7.4 (3.8–33.4)	8.8 (2.4–41)	0.59
Albumin, median (IQR), g/L	36.2 (34.2–45)	40.7 (30.9–46.2)	0.002
GI symptoms, *n* (%)	7/13 (53.8%)	5/23 (21.37%)	0.04
FFMI, median (IQR), kg/m^2^	13.9 (11.2–14.2)	16.7 (14.1–18.7)	<0.0001

BMI: body mass index. lcSSC: limited cutaneous systemic sclerosis. dcSSC: diffuse cutaneous systemic sclerosis. mRSS: modified Rodnan skin score. BMD: bone mineral density. TBS: trabecular bone score. Hb: hemoglobin. 25(OH)D: 25(OH) vitamin D. Ca: calcium. Ph: phosphorus. PTH: Parathyroid hormone. ALP-b: bone alkaline phosphatase. SIBO: small intestine bacterial overgrowth. FFMI: free fat mass index. *Smoke: at least one cigarette a day. **Alcohol consumption: light to moderate drinking that considered fewer than 60 g of pure alcohol per day in men and fewer than 40 g in women (WHO 2000).

**Table 2:** Correlation between fat free mass index (FFMI) and bone parameters in SSc.

	Bone Parameters	r	*p*
**FFMI**	Weight, kg	0.61	<0.0001
	BMI, kg/m^2^	0.65	<0.0001
	Legs BMD, g/cm^2^	0.44	0.006
	Total trunk BMD, g/cm^2^	0.52	<0.001
	Lumbar spine, g/cm^2^	0.49	0.002
	Ribs BMD, g/cm^2^	0.44	0.006
	Pelvis BMD, g/cm^2^	0.46	0.004
	Total femur BMD, g/cm^2^	0.42	<0.01
	TBS	0.42	<0.01
	Hb (g/dL)	0.50	<0.001
	PTH (ng/L)	0.33	0.04
	25(OH)D (ng/mL)	0.38	0.02
	Albumin (g/L)	0.40	<0.01

FFMI: fat free mass index. BMI: body mass index. BMD: bone mineral density. TBS: trabecular bone score. Hb: hemoglobin 25(OH) D: 25(OH) vitamin D. PTH: Parathyroid hormone. r-Spearman’s correlation coefficient, *p* ≤ 0.05.
